# Supplementary material for: Dairy Cows Grazing Plantain-Based Pastures Have Increased Urine Patches and Reduced Urine N Concentration That Potentially Decreases N Leaching from a Pastoral System
Source: Animals (Basel). 2023 Feb 2;13(3):528. doi: 10.3390/ani13030528 (PMC9913302; doi:10.3390/ani13030528)
Supplement: Supplementary file 1 [file animals-13-00528-s001.zip › animals-2117894-supplementary.pdf]

Equation S1. Estimation of the area covered by urine patches

Urine patch area (m^2/cow/day) = daily urination volume (m^3) ÷ urine column (m) × number of urination per day [15], assuming a urine column to be 9 mm [2].

Equation S2. Estimation of urinary nitrogen (UN) load

N load at urine patches (kg/ha) = UN excretion (kg) ÷ urine patch area (ha) [15],

where, UN excretion (kg) = urine volume (L) × UN concentration (kg/L).
